# Long COVID – eine neue Herausforderung in der Medizin: Fokus auf Schwangerschaft und Stillzeit

**DOI:** 10.1007/s41974-023-00250-5

**Published:** 2023-02-02

**Authors:** Emilie Han, Mariann Gyöngyösi

**Affiliations:** Abteilung für Kardiologie, Universitätsklinik für Innere Medizin II, Medizinische Universität Wien, Währinger Gürtel 18–20, 1090 Wien, Österreich

**Keywords:** Post-COVID-Syndrom, Menstruation, Schwangerschaft, Stillzeit, COVID-Impfung, Post-acute sequelae of COVID-19, Menstruation, Pregnancy, Lactation, COVID vaccine

## Abstract

Long COVID wurde als eine neue Multiorganerkrankung beschrieben, die bei Frauen häufiger auftritt als bei Männern. Schwangere und stillende Frauen sind eine spezielle Untergruppe von Patienten, die bei einer Long-COVID-Erkrankung zu berücksichtigen sind, da bisher die Datenlage nur gering ausfällt. Menstruationsveränderungen werden häufig während oder nach einer akuten Erkrankung mit dem Coronavirus-2019 (COVID-19) beobachtet. Einige Studien stellen zudem einen Zusammenhang zwischen geringen Veränderungen der Zykluslänge und einer COVID-Impfung dar. Schwangere Frauen, die eine symptomatische Infektion mit dem schweren-akuten-Atemwegssyndrom-Coronavirus Typ 2 (SARS-CoV‑2) hatten, können ein höheres Risiko für Komplikationen in der Schwangerschaft, wie Frühgeburt oder Präeklampsie, haben. Darüber hinaus sind mehr Studien notwendig, um die Auswirkungen einer vertikalen Übertragung zu beurteilen. Das wirksamste Mittel gegen die Pandemie sind die verfügbaren COVID-Impfstoffe, da sie eine Infektion verhindern, aber auch Long-COVID-Symptome lindern können. Impfstoffe haben sich sowohl bei schwangeren als auch bei stillenden Frauen als sicher und wirksam erwiesen. Ziel dieses Artikels ist es, die aktuell verfügbaren Daten zu Long COVID bei schwangeren und stillenden Frauen darzustellen und die Risikofaktoren und therapeutischen Möglichkeiten aufzuzeigen.

## Abstract (en)

Long COVID (coronavirus disease) has been described as a new multi-organ disease, which appears to be more prevalent in women than in men. Pregnant and breastfeeding women are a special subgroup of patients to consider with long COVID, as only scarce data have been collected to date. Menstrual changes are commonly observed during or after COVID-19; some studies also attribute slight changes of cycle length to previous inoculation against the virus. Pregnant women who have a symptomatic infection with severe acute respiratory syndrome coronavirus type 2 (SARS-CoV-2) are at a higher risk for adverse outcomes and pregnancy-associated complications. Moreover, more and robust data are required to evaluate vertical transmission. COVID vaccines are the most effective tool against the pandemic, as they prevent infection, but also appear to be able to ease long COVID symptoms. Vaccines have been proven safe and effective in both pregnant and breastfeeding women. This article aims to present current data on long COVID in pregnant and breastfeeding women and elucidate risk factors and possible treatment options.

Nach einer Infektion mit dem schweren-akuten-Atemwegssyndrom-Coronavirus Typ 2 (SARS-CoV-2)-Virus erholt sich der Großteil der Genesenen vollständig von der Coronarvirus(COVID)-19-Erkrankung, jedoch kann es in ungefähr 3–37 % der Fälle durch eine lange oder inkomplette Rekonvaleszenz der Infektion zu der sogenannten Long-COVID-Erkrankung kommen [1, 2]. Diese Patient:innen mit einem Long-COVID-Syndrom klagen über verschiedene Symptome, wie zum Beispiel Fatigue, Erschöpfung, Kopfschmerzen, Schwäche, Husten, Brustschmerzen, Palpitationen oder eine Einschränkung der täglichen Aktivitäten, eine schnell auftretende Tachykardie oder einen Abfall des Blutdrucks, auch posturales Tachykardiesyndrom (POTS) genannt [3]. Einige Patient:innen befinden sich nach einer akuten Infektion über Monate oder sogar bis zu einem Jahr im Krankenstand [4]. Durch die zahlreichen organverbundenen Symptome wird das Long-COVID-Syndrom als eine neue Multiorganerkrankung bezeichnet.

Auffallend ist, dass Männer tendenziell häufiger an COVID-19 erkranken und auch eher schwerwiegendere Verläufe haben [5, 6]. Währenddessen besteht der überwiegende Anteil der Long-COVID-Patient:innen aus Frauen, welche oftmals einen nur milden Verlauf der akuten Erkrankung aufweisen [7]. Eine besondere Patientengruppe stellen dabei schwangere und stillende Frauen dar, da die Datenlage in dieser Population gering ist. Dieser Bericht soll einen Überblick zur derzeitigen wissenschaftlichen Literatur bei Long COVID bei schwangeren und peripartalen Frauen schaffen.

## Pathophysiologie von Long COVID und die geschlechterspezifischen Charakteristika

Folgende Hypothesen bestehen bezüglich der Pathophysiologie von Long COVID: (i) SARS-CoV‑2 vermittelt über direkte Zytotoxizität länger andauernde Zellschäden und Störungen im Zellmetabolismus, (ii) die durch die akute Erkrankung ausgelöste Hyperinflammation und Zytokinstürme haben einen weiteren Einfluss im Körper, (iii) die physiologische Reaktion der Virusabwehr führt zu einer vermehrten Bildung gegenregulatorischer Hormone und zur Induktion verschiedener Signalwege in den jeweiligen Organsystemen [8].

Frauen haben einen gewissen immunologischen Schutz vor Infektionen, da mehrere immunregulierenden Gene auf dem X‑Chromosom lokalisiert sind [9]. Diese Gene können sowohl an der angeborenen als auch an der adaptiven Immunantwort beteiligt sein und somit den höheren immunologischen Schutz bei Frauen erklären [10, 11]. ACE2 ist ein membrangebundener Rezeptor, der ebenso von auf dem X‑Chromosom lokalisierten Genen exprimiert wird, hauptsächlich in Lunge, Niere, Herz und Darm vorkommt [12] und als ein Rezeptor für SARS-CoV‑2 zum Eintritt in die Zelle fungiert [13]. Demnach sollten weibliche Patienten anfälliger auf eine SARS-CoV-2-Infektion reagieren und heiklere Verläufe haben [14]. Allerdings berichteten mehrere Studien über niedrigere SARS-CoV-2-Morbiditäts- und Mortalitätsraten bei weiblichen im Vergleich zu männlichen Patienten [15, 16]. Ein schwerwiegenderer Verlauf von COVID-19 bei Männern könnte auf eine höhere Inzidenz atherosklerotischer Risikofaktoren bei männlichen im Vergleich zu weiblichen Patient:innen zurückzuführen sein [17], da diese (arterielle Hypertonie, Diabetes, Dyslipidämie) in Studien bereits als Risikofaktor für mehr Intensivaufenthalte gezeigt wurden [18]. Die Hypothese, dass durch kardiovaskuläre Risikofaktoren die geschlechterspezifischen Unterschiede einer SARS-CoV-2-Infektion geklärt werden können, wird jedoch nicht einheitlich in der Literatur so betrachtet. Eine andere Studie konnte diese Ergebnisse zum Beispiel nicht vollständig bestätigen, da festgestellt wurde, dass der Unterschied in der Mortalität nicht vollständig durch die höhere Prävalenz von Komorbiditäten erklärt werden konnte [16].

Rein proportional sollten mehr männliche Patienten ein Long-COVID-Syndrom haben, da COVID-19 häufiger bei Männern diagnostiziert wird [19]. Jedoch sind fast 70 % der Patienten mit Long COVID weiblich und in einer Studie aus Irland wurde als einziger Prädiktor für eine erhöhte Punktzahl auf der modifizierten Borg-Dyspnoe-Skala (MBS) das weibliche Geschlecht als signifikanter Faktor gefunden [20]. Während einer Schwangerschaft verändern sich außerdem die physiologischen Prozesse im Körper der Frau. Das Risiko einer schwangeren Frau, bei einer akuten COVID-19-Infektion hospitalisiert zu werden, ist gegenüber der restlichen Bevölkerung erhöht; Gründe könnten einerseits die Immunsuppression, andererseits die verringerte Lungenkapazität während der Schwangerschaft sein [21]. In einer Metaanalyse, die 192 Studien und insgesamt mehr als 67.000 Frauen inkludierte, fanden die Autoren, dass das Risiko für eine Frühgeburt bei schwangeren Frauen mit einer akuten SARS-CoV-2-Infektion dreifach so hoch ist wie bei COVID-19-negativen Frauen [22]. Auch das Risiko für eine Präeklampsie ist erhöht, wenn gleichzeitig zur Schwangerschaft eine Thrombozytopenie besteht [23]. Außerdem scheint SARS-CoV‑2 direkt die Plazenta anzugreifen und infizieren zu können, was wiederum zu einer plazentaren Insuffizienz und darauffolgend einer perinatalen Asphyxie führen kann [24].

## Long COVID und Menstruationsveränderungen

Eine Frau, die bis dato reguläre Zyklusabstände hatte, berichtete in einer anekdotischen Erzählung von Veränderungen ihres Menstruationszyklus nach COVID-19, dass sie beispielsweise nur fünf Menstruationszyklen in einer Zeitspanne von zwei Jahren hatte [25]. Die Ergebnisse von drei Studien aus einem systematischen Review berichten von verringertem Menstruationsvolumen und einem verlängerten Zyklus als häufigste Änderungen als Folge einer SARS-CoV-2-Infektion. Die Ergebnisse deuten auch darauf hin, dass der Schweregrad von COVID-19 bei Veränderungen des Menstruationszyklus keine Rolle spielt [26]. Weitere Forschungsgruppen haben sich bereits mit diesem Thema auseinandergesetzt. Es stellte sich heraus, dass von 524 Frauen nach einer COVID-19-Erkrankung rund 19 % Veränderungen der Menstruation hatten, wobei knapp die Hälfte dieser aus Hypomenorrhö bestand [27]. In einer globalen Fragebogenstudie der Patient-Led Research Collaborative (einer Gruppe an Personen, die selbst an Long COVID leiden) zeigte sich bei circa 5 % der teilnehmenden Frauen eine Frühmenopause, während weitere 5 % von Zwischenblutungen berichteten, obwohl sie bereits nach der Menopause waren [28].

Warum eine Infektion mit SARS-CoV‑2 zu Menstruationsveränderungen führt, ist noch nicht geklärt, ebenso wenig, warum Impfungen gegen das Virus eine ähnliche Symptomatik hervorbringen können. Dazu existieren Daten aus den Vereinigten Staaten: In einer Studie mit fast 4000 Teilnehmern konnte man zeigen, dass Impfungen gegen COVID-19 mit einer geringen Änderung der Zykluslänge, aber nicht der Menstruationslänge assoziiert sind [29]. In einer Metaanalyse mit fast 40.000 geimpften Frauen fanden die Autoren einen Zusammenhang zwischen höherem Alter und verstärkter Menstruationsblutung bei prämenopausalen Frauen (18–45 Jahre) mit lateinamerikanischer Herkunft. Ebenso waren vorangegangene Schwangerschaften und Geburten oder eine bereits vorher diagnostizierte gynäkologische Erkrankung (z. B. Endometriose, Myome, polyzystisches Ovarialsyndrom) mit einem höheren Risiko für stärkere Blutungen verbunden [30].

## Long COVID bei schwangeren Frauen

Derzeit sind noch keine epidemiologischen Zahlen vorhanden, die die Anzahl der von Long COVID betroffenen schwangeren Frauen erläutern. Ein wesentlicher Faktor, der die Diagnose von Long COVID bei schwangeren und postpartalen Frauen erschwert, sind die ähnlichen Beschwerden, die von sowohl Patient:innen mit Long COVID als auch schwangeren Frauen berichtet werden. Zum Beispiel zählen Fatigue und/oder Dyspnoe zu den Leitsymptomen von Long COVID [31] und sind gleichzeitig auch bei einer normalen Schwangerschaft in vielen Frauen ausgeprägt. Während der peripartalen Phase kann die Verwendung eines Symptomtagebuchs eine frühzeitige Erkennung neuer oder ein Aggravieren bereits vorhandener Symptome ermöglichen. Eine vorhergehende Long-COVID-Diagnose schließt die Entwicklung einer neuen schwangerschaftsassoziierten Erkrankung nicht aus und daher sollten alle neu auftretenden Symptome detailliert evaluiert werden, bevor sie einem vorbestehenden Long-COVID-Syndrom zugeschrieben werden [32].

Es ist bereits bekannt, dass chronische Erschöpfung nach Infektionskrankheiten, insbesondere nach Viruserkrankungen, zur Entwicklung einer myalgischen Enzephalomyelitis (ME) oder eines chronischen Fatiguesyndroms (CFS) führen kann. In einem Preprint einer aktuellen Studie wurde über das häufigere Auftreten von Fatigue nach einer symptomatischen SARS-CoV-2-Infektion bei Schwangeren verglichen mit seronegativen und seropositiven, aber asymptomatischen Schwangeren bei der Geburt berichtet. Zudem waren das Risiko, an Fatigue zu leiden, und die zeitliche Dauer mit der Schwere der COVID-19-Erkrankung assoziiert [33]. In einer Metaanalyse, die 10.000 schwangere Frauen mit bestätigter COVID-19 inkludierte, wurde eine Rate der vertikalen Übertragung von 5,3 % ermittelt und ein erhöhtes Risiko für Frühgeburten und notwendige Kaiserschnitte gefunden [34].

## Die Impfung schützt vor Long COVID

In einer rezent publizierten Metaanalyse mit insgesamt über einer Million Patient:innen aus 18 Studien fand man, dass die COVID-Schutzimpfung das Risiko für Long COVID unabhängig vom Zeitpunkt der Impfung verringert [35]. Das heißt, selbst nach einer bereits abgelaufenen COVID-19-Erkrankung stellt das Impfen danach noch einen Schutzfaktor für Long COVID dar. Was die Gründe dafür sind, ist derzeit noch nicht komplett erforscht. Hypothesen lauten, dass die Impfung bei einer aus dem Lot geratenen Immunreaktion bzw. Autoimmunreaktion nach der SARS-CoV-2-Infektion einen sogenannten „Reset“ im Immunsystem bewirken kann, um so die normale Funktion wiederherzustellen [36]. Die aktuelle Forschung beschäftigt sich mit den potenziellen Wirkmechanismen der COVID-Impfstoffe auf Long COVID, wie die Impfung (i) die restlichen, sich noch im Körper befindenden Coronaviren oder deren Fragmente beseitigen, (ii) durch einen sogenannten „Neustart“ das entgleiste Immunsystem wiederherstellen oder (iii) falsch-funktionierende Immunprozesse unterbinden und beheben könnte. Außerdem muss man die geschlechtsspezifischen Unterschiede bezüglich Impfstofftoleranz und -wirksamkeit beachten, da Frauen aufgrund der höheren stimulierten Interferonaktivität im Allgemeinen eine stärkere Impfreaktion besitzen; daher könnten Frauen durch die Impfung einen längeren und stärkeren Schutz gegen Viren haben [37].

## COVID-Impfempfehlung während der Schwangerschaft und Stillzeit

In Fallberichten wurde über eine Infizierung von plazentaren Zellen berichtet [38, 39], was jedoch nicht mit einer fetalen Infektion gleichzusetzen ist. Jedoch können virale Infektionen lang anhaltende schädliche Auswirkungen auf den Fetus haben. Während der Schwangerschaft befindet sich der mütterliche Körper abwechselnd in einem pro- (im ersten und dritten Trimester) und anti-inflammatorischen (im zweiten Trimester) Zustand, so kann durch eine ausgeprägte mütterliche Entzündungsreaktion auf das SARS-CoV-2-Virus das fetale Wachstum beeinflusst werden [40]. In einer Studie mit über 1300 schwangeren Frauen aus Großbritannien wurden keine Unterschiede in Komplikationen während der Geburt oder sonstigen perinatalen Endpunkten gefunden [41]. Fernerhin greift die COVID-Schutzimpfung, trotz anfänglicher Befürchtungen in der Bevölkerung, nicht in die Fertilität bei Frauen oder Männern ein und ist in dieser Hinsicht unbedenklich [42]. Vielmehr könnte es im Verlauf einer COVID-19-Erkrankung zu einer Schädigung der Reproduktionsorgane, durch eine NLRP3-Inflammasom-Hyperaktivierung und ein prokoagulatorisches Milieu ähnlich wie bei einem Mastzellaktivierungssyndrom, kommen [43–45].

Nach dem Wissensstand von Oktober 2022 empfiehlt das Robert Koch-Institut (RKI) die COVID-Impfung allen Frauen, die einen aktuellen Kinderwunsch haben. Auch bereits schwangere Frauen sollen die vollständige COVID-Impfung inklusive Auffrischungsdosis (dritte Impfdosis, vorzugsweise mit einem an Omikron adaptierten mRNA-Impfstoff) erhalten [46]. In einer Stellungnahme der Österreichischen Gesellschaft für Gynäkologie und Geburtshilfe (ÖGGG) zu Booster-Impfungen für Schwangere und stillende Frauen wird eine weitere Auffrischungsdosis (4. Impfung) in der Schwangerschaft als sinnvoll erachtet, insbesondere im Hinblick auf mögliche erneut stärkere Infektionsgeschehen [47].

Es können überdies nach einer erfolgten Immunisierung die mütterlichen Antikörper via diaplazentaren Transfer oder über die Muttermilch beim Stillen an das Baby weitergegeben werden [48]. Zu den Effekten der auf Omikron adaptierten Impfstoffe auf schwangere und stillende Frauen ist die derzeitige Datenlage noch unzureichend, um gesicherte Aussagen treffen zu können.

Die COVID-Schutzimpfung ist grundsätzlich aufgrund der Risiko-Nutzen-Abwägung zu jedem Zeitpunkt in der Schwangerschaft für sowohl die schwangere Frau als auch ihr ungeborenes Baby sicher und schützt vor der SARS-CoV-2-Infektion, die zu schwerwiegenden Komplikationen für Mutter und Kind führen kann [41]. Jedoch gilt die Empfehlung derzeit, dass bevorzugt das erste Trimenon abgewartet werden sollte, da in der frühen Phase der Schwangerschaft Spontanaborte häufig sind und diese durch eine Empfehlung für die Impfung später in der Schwangerschaft weniger leicht kausal mit der Impfung in Verbindung gebracht werden könnten.

## Risikofaktoren und Therapie für Long COVID in der Schwangerschaft

Die Risikofaktoren für einen schwerwiegenden Verlauf bei Schwangeren sind deckend mit denen in der restlichen Bevölkerung, dazu gehören unter anderem Diabetes mellitus und Übergewicht [49]. Derzeit steht noch nicht fest, ob Long COVID einen Risikofaktor für eine Schwangerschaft darstellt oder ob eine Schwangerschaft kurzzeitig nach einer SARS-CoV-2-Infektion bzw. eine Infektion während der Schwangerschaft mit einem höheren Risiko, an Long COVID zu erkranken, verbunden ist. Jedoch fand eine brasilianische Fall-Kontroll-Studie mit 84 schwangeren Frauen mit PCR-bestätigter COVID-19, dass 80 % der Studienteilnehmer an Long-COVID-Symptomen litten, und bei einem Drittel der Frauen persistierten die Symptome auch noch postpartal [50]. Auch in einer größeren retrospektiven Studie mit 14.104 Patientinnen stellte man fest, dass maternale Mortalität und schwerwiegende Schwangerschaftskomplikationen (z. B. hypertensive Störungen, postpartale Blutungen) bei SARS-CoV-2-infizierten Frauen 40 % häufiger auftraten als bei seronegativen Frauen [35].

Bei der Therapie von Long COVID bei Schwangeren gibt es derzeit keine Unterschiede zur Therapie der restlichen Bevölkerung. Ein etwaiges Medikament, das kausal helfen könnte, wurde bisher noch nicht entwickelt; daher beruht die Therapie auf der symptomatischen Behandlung der Erkrankung. Mögliche Therapieoptionen für Long COVID beinhalten unter anderem, mit einem individualisierten, strukturierten und schrittweise ausgebauten Aktivitäts- und Rehabilitationsprogramm zu beginnen; Strategien zu entwickeln, um die vorhandene Energie möglichst effizient zu nutzen; ein gesundes Ernährungsmuster zu entwickeln und auf ausreichende Flüssigkeitszufuhr zu achten und vorhandene Grunderkrankungen, wie Schmerzen, Schlafstörungen und/oder Stimmungsprobleme, mit einem multidisziplinären Team aus Spezialist:innen zu behandeln [51].

## Konklusion

Die Forschungslage zu Long COVID in der Schwangerschaft und Stillperiode steckt noch sinngemäß in den Kinderschuhen, da selbst die Literatur zu COVID-19 bei schwangeren Frauen gering ausfällt. Es benötigt zum einen mehr Zeit und eine größere Ansammlung an Daten, um gefestigtere Aussagen über Long COVID in dieser Patientengruppe tätigen zu können, zum anderen auch eine zunehmende Förderung der Erforschung der pathophysiologischen Prozesse von COVID-19 und wie SARS-CoV‑2 das endokrinologische und immunologische System des Körpers beeinflusst.

## Fazit für die Praxis

- Frauen, die während der Schwangerschaft an Coronavirus-19 (COVID-19) erkranken, haben ein höheres Risiko für schwerwiegende Schwangerschaftskomplikationen, Frühgeburten und Präeklampsie.
- Menstruationsveränderungen können sowohl nach COVID-19 als auch nach einer COVID-Schutzimpfung auftreten.
- Derzeit existiert noch keine kausale Therapie gegen Long COVID, daher kann man nur mit physikalischen und psychischen Rehabilitationsmaßnahmen versuchen, die Symptome in schwerwiegenden Verläufen zu lindern.
- Die COVID-Schutzimpfung (unabhängig vom Zeitpunkt der Impfung) verringert das Risiko für das Long-COVID-Syndrom.
- Die Impfung ist unbedenklich für sowohl die Fertilität als auch die Sicherheit der Schwangeren oder ihres Kindes; auch die Auffrischungsimpfung (dritte Impfdosis mit vorzugsweise bivalenten Impfstoffen) wird ausdrücklich empfohlen.
- Frauen mit Kinderwunsch sollten optimal vor der Konzeption bereits vollständig geimpft sein, ansonsten ist eine COVID-Schutzimpfung im zweiten und dritten Trimenon der Schwangerschaft möglich. Dabei wurde im Vergleich zu Frauen, die nicht in diesem Zeitraum geimpft wurden, keine höhere Rate an Komplikationen während der Schwangerschaft beobachtet.
